# Noisy Breathing in an Infant: A Case Report

**DOI:** 10.7759/cureus.13015

**Published:** 2021-01-30

**Authors:** Charlie J Sang, Camden Hebson

**Affiliations:** 1 Internal Medicine/Pediatrics, University of Alabama at Birmingham, Birmingham, USA; 2 Division of Pediatric Cardiology, Department of Pediatrics, University of Alabama at Birmingham, Birmingham, USA

**Keywords:** double aortic arch, vascular ring, congenital heart disease, tracheal stenosis

## Abstract

The diagnosis of vascular rings is challenging and may be delayed as symptoms overlap with more common conditions associated with childhood. Underlying genetic associations of this condition remain largely undiscovered. In this report, we present a patient with a double aortic arch and highlight the importance of diagnostic imaging. We also engage in a review of the important genetic considerations.

## Introduction

Vascular rings account for approximately 1% of all congenital heart defects [1] and often go undetected until sufficiently severe symptoms arise. Autopsy studies have shown that 3% of people have a congenital arch anomaly, with two-thirds of these cases being previously undiagnosed [1]. Symptoms occur due to compression of the trachea and esophagus [1], with severe disease presenting during infancy with stridor that worsens with feeding and improves when calm or asleep [2]. Infants may also experience a failure to thrive, as increased work of breathing limits intake and increases caloric needs [2]. In milder forms, identification may be delayed, and patients may be misdiagnosed with asthma or gastroesophageal reflux [1,2]. Due to the often seemingly familiar presentation of vascular rings, it is imperative to maintain a high index of suspicion for the diagnosis. We present a case of double aortic arch and tracheal stenosis in an infant with acute hypoxic respiratory failure and demonstrate the importance of echocardiography and CT in the diagnosis and surgical planning. We also review the important genetic considerations.

## Case presentation

A full-term two-month-old female infant was brought in with respiratory distress. Her prior history was notable for noisy breathing since birth, a symptom that waxed and waned with feeding and sleep, respectively. She had been evaluated by otolaryngology at one month of age, and the examination had revealed mild laryngomalacia at the least with foreshortened aryepiglottic folds and redundant mucosa of the arytenoids. Prior to her current presentation, she had experienced several days of worsening tachypnea.

Upon initial presentation, she was afebrile, with a heart rate of 167 bpm, blood pressure of 102/74 mmHg, respiratory rate of 62 bpm, and oxygen saturation of 96% on nasal continuous positive airway pressure. The facial exam was notable for a short, upturned nasal tip and retrognathia. She had marked congestion and was in respiratory distress, with coarse breath sounds bilaterally, as well as inspiratory and expiratory stridor. She had a regular rate and normal rhythm, without the presence of a murmur. Proximal and distal pulses were 2+ bilaterally. The abdomen was soft, without organomegaly. There were no obvious skeletal abnormalities. She was otherwise awake and alert. She moved her extremities symmetrically and with a normal tone.

**Figure 1:**
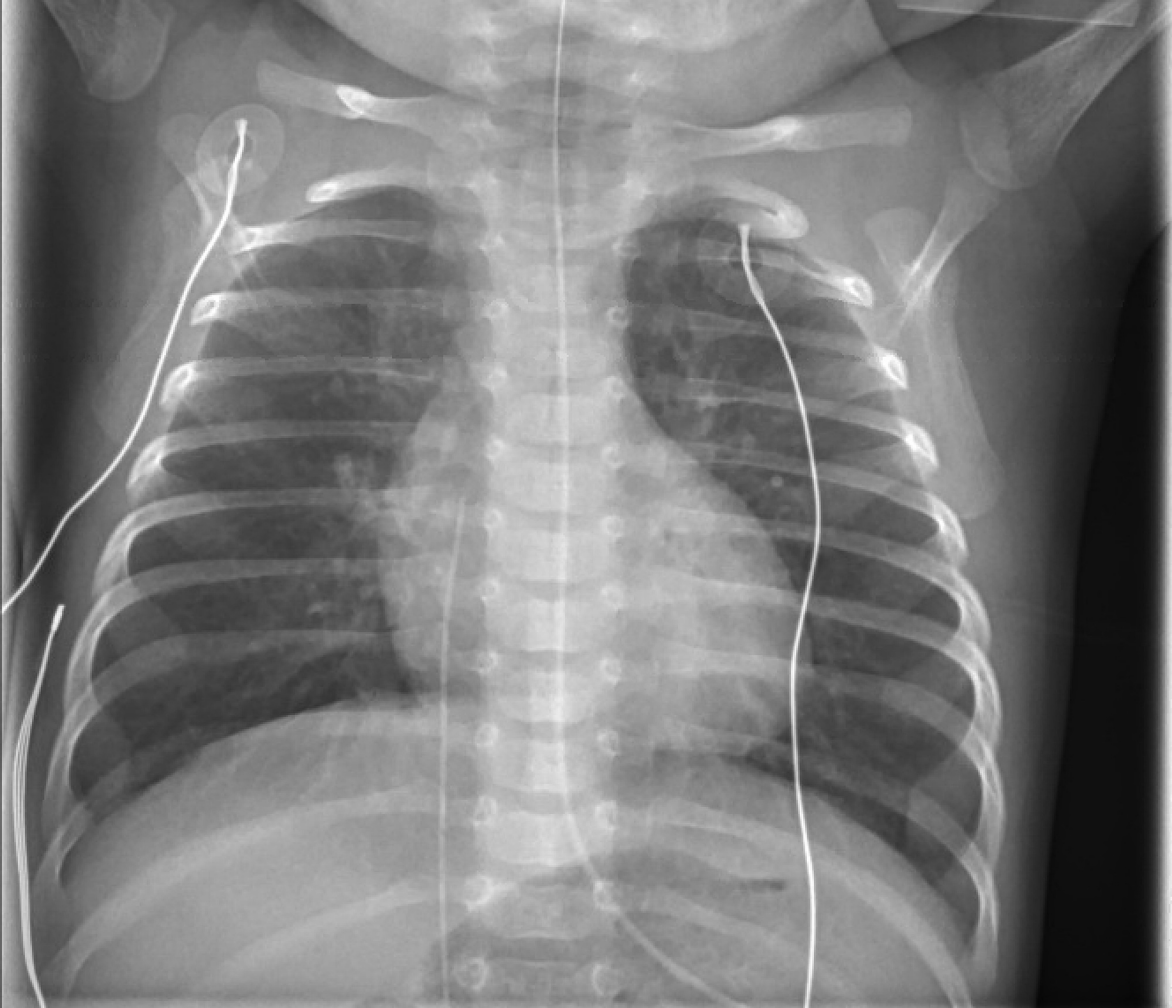
Chest radiograph The tracheal air column is poorly visualized. Lungs are well-expanded. No pneumothorax or consolidation is identified. Heart size is normal. Aortic arch sidedness is indeterminant

Initial lab work was notable for a white blood cell count of 17.97 (normal range: 6.00-13.25 x 10^3^ u/L) without bandemia, hematocrit of 35, and platelet count of 927 (normal range: 140-440 x 10^3^ u/L). A viral panel was positive for the respiratory syncytial virus (RSV). Chest X-ray revealed well-expanded lungs, without pleural effusion, pneumothorax, or focal consolidation. The heart size was grossly normal. The aortic arch sidedness was indeterminant. The tracheal air column was poorly visualized (Figure 1). Despite appropriate and intensive initial care, she developed worsening respiratory distress over the following days, eventually requiring high-frequency oscillatory ventilation, systemic steroids, intravenous sedation, and paralytics.

**Figure 2:**
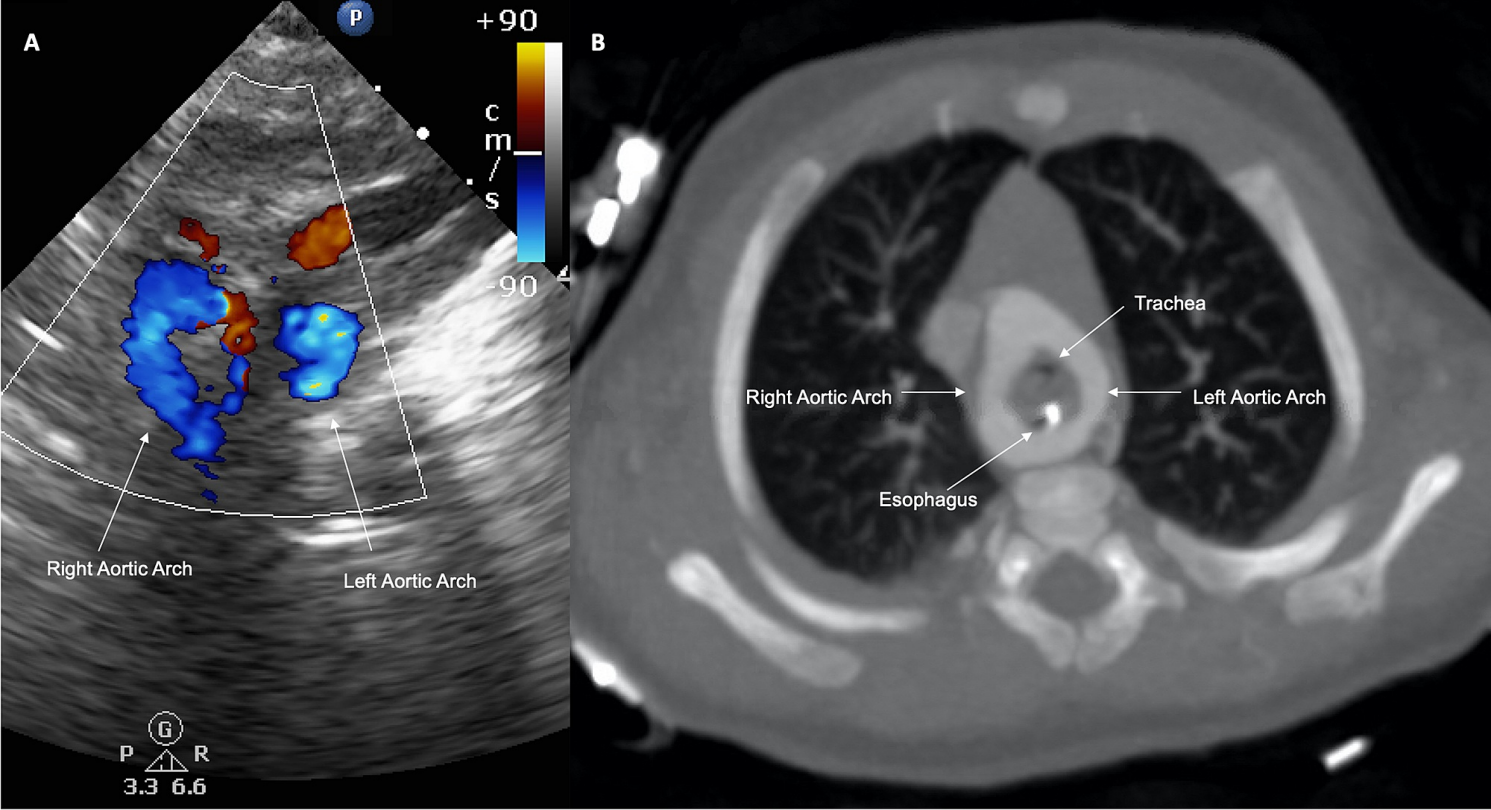
Transthoracic echocardiogram and CT angiography (A) Transthoracic echocardiogram, suprasternal short axis; color Doppler images suggestive of the double aortic arch. (B) CT angiography, axial plane; there is a right dominant double aortic arch encircling the trachea and esophagus CT: computed tomography

**Figure 3:**
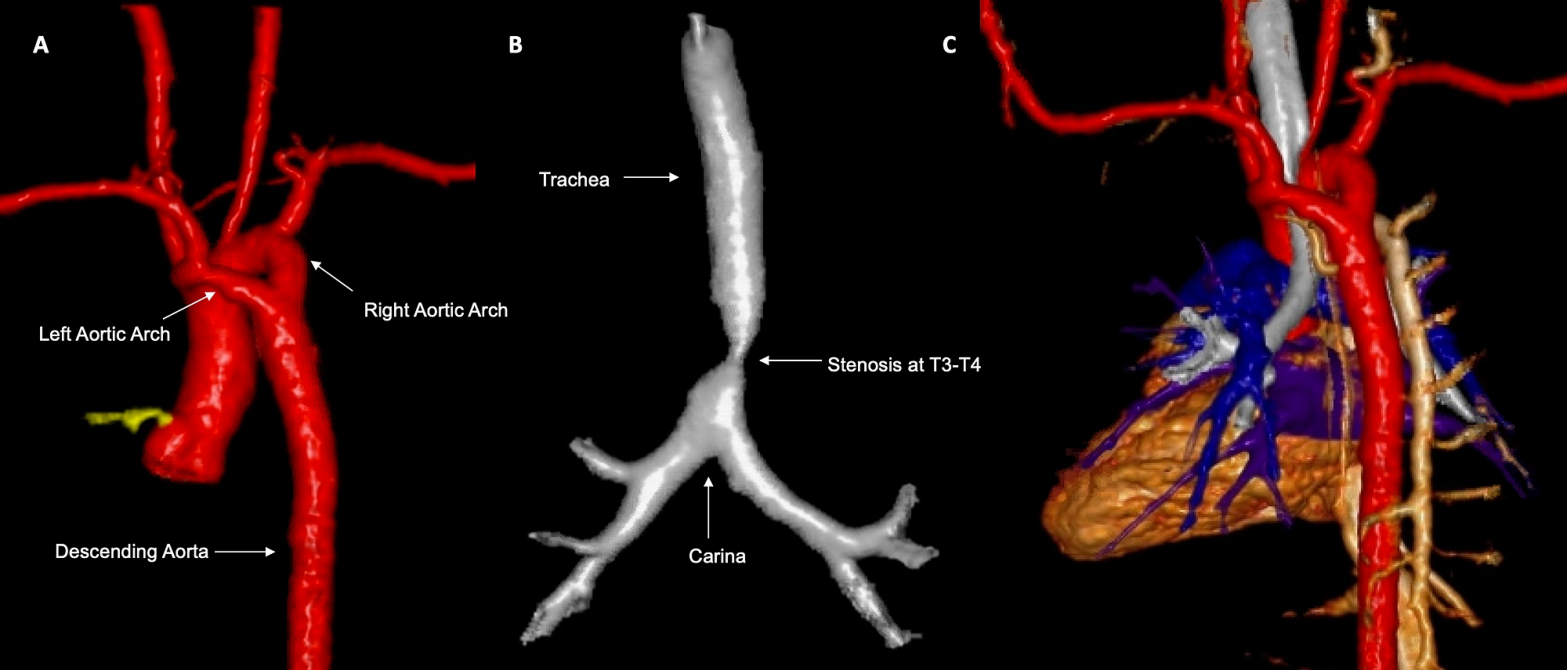
3D reconstruction of the CT (A) 3D reconstruction of great vessels showing the double aortic arch from the posterior view. (B) 3D reconstruction of airway showing severe distal tracheal stenosis. (C) Complete 3D reconstruction showing full anatomical positions CT: computed tomography

Given her deteriorating status and escalation of care, an echocardiogram was obtained, which raised concern for a double aortic arch (Figure 2A). CT angiography confirmed the right dominant double aortic arch (Figure 2B), with 3D reconstruction delineating marked narrowing of the distal trachea due to vascular compression (Figure 3A, Figure 3B, Figure 3C). There were no additional aortic or branch abnormalities identified. Given her aortic arch defect and dysmorphic features, a genetic consultation was requested. Microarray analysis revealed a 4.85 Mb duplication at 10q11.22q11.23x3. This finding was judged to be a variant of uncertain significance.

The patient's case was discussed at the weekly multi-disciplinary pediatric and adult congenital cardiovascular conference. The decision was made to proceed with surgical intervention, and informed consent was obtained from the family. Following a period of stabilization and recovery from RSV, the patient was brought to the operating room. She underwent a left posterolateral thoracotomy. The left subclavian artery, right and left aortic arch, and descending aorta were dissected out. Once identified, the segment of the left arch distal to the left subclavian artery was test occluded without any change in blood pressure or oxygen saturation. The distal left arch was then divided and each end was oversewn. Fibers between the aortic arch and the esophagus were divided to ensure that the space was adequately open. Her postoperative care was routine and uneventful. At the two-month follow-up visit, she was thriving with mild stridor, which is expected to improve over the next several months.

## Discussion

Respiratory complaints are among the most common difficulties encountered by pediatric providers. The differential diagnosis generally includes the respiratory, cardiovascular, and gastrointestinal systems, with underlying infectious, inflammatory, or anatomical etiologies commonly considered. For the present case, viral bronchiolitis exacerbated by underlying laryngomalacia was initially thought to be the most likely cause of the severe respiratory failure. Other diagnoses that should be considered in similar patients include primary airway anatomic disease (granulomas, webs, cysts, clefts, vocal cord dysfunction), infectious/inflammatory conditions (croup, epiglottis, retropharyngeal abscess, tracheitis), presence of an airway foreign body, pulmonary artery sling, and of course vascular rings.

Vascular rings represent both a failed (double aortic arch) or aberrant (right aortic arch) regression of one of the paired dorsal aortas during embryologic development [1]. In a double aortic arch, the vascular ring forms anteriorly via the bifurcation of the ascending aorta. The two arches course leftward and rightward of the trachea and reform posteriorly to the esophagus to form the descending aorta, resulting in a ring [1]. The double aortic arch, along with the right aortic arch with aberrant left subclavian artery, makes up the large majority of vascular rings [3,4]. 

Identification of vascular rings may be delayed (even into adulthood) in patients with mild symptoms or sufficiently overlapping symptoms with more common pathologic conditions [1,2,5]. A successful diagnosis requires the clinician to remain open-minded and to continually reassess potential diagnoses, particularly in the setting of an insufficient response to treatment [1]. In our patient’s case, escalation of ventilatory support and inability to wean from mechanical ventilation was the major stimulus to obtain further thoracic imaging.

In patients with respiratory symptoms, chest X-rays are often the initial imaging modality; however, these are poor screening exams for vascular rings, with some studies reporting diagnostic yields of <5% [6]. Chest X-rays are often obtained to assess for etiologies other than vascular rings; therefore, if close scrutiny of the tracheal air column is not performed, indirect findings of aortic arch abnormalities may be missed [6]. Additionally, plain radiographic findings may be limited due to interference with the thymic shadow or technical issues related to thoracic rotation [7]. For the present case, the initial X-ray demonstrated poor visualization of the tracheal air column, which may be an indirect clue to an arch abnormality.

By contrast, barium esophagography offers improved sensitivity, detecting characteristic esophageal indentations in >90% of affected patients [6]. While not performed in our patient, barium esophagography has its place in the diagnostic algorithm, specifically when other evaluations such as echocardiography are not readily available for patients presenting with suspicious symptoms. As for echocardiography, one diagnostic weakness is its inability to visualize atretic segments of vascular rings [8]. Furthermore, in a double aortic arch, the left arch is often in an appropriate position and may give the impression of normal anatomy if detailed sweeps are not obtained. Differences in diagnostic yield for echocardiography have been reported, and variations in imaging techniques and protocols likely explain much of this discrepancy [6,9]. Therefore, three-dimensional imaging, either through cardiac CT or cardiac MRI, is often obtained to confirm the diagnosis or if suspicion remains after seemingly normal echocardiographic findings [7].

Treatment is surgical and offers an extremely favorable long-term prognosis [3,10]. Indications for surgery include symptoms produced by the vascular ring compression (stridor, feeding difficulties). There are patients diagnosed with vascular rings who are asymptomatic, and we do not advocate for surgery in this scenario. The surgical plan itself depends on the anatomy and can involve ligation and division of the non-dominant aortic arch in the setting of a double aortic arch, and ligamentum release in the setting of less complex anatomy [11]. Additionally, increased complexity regarding the surgical planning of vascular rings may arise in circumflex aortic arches or if there is concomitant compression from the descending thoracic aorta. These anomalies may require aortic translocation for relief of tracheal or bronchial compression [12,13].

The field of cardiogenetics represents a rapidly expanding discipline, with significant implications for patients and their families. In general, parallel sequencing and microarrays are of low diagnostic yield in isolated congenital heart disease, with new diagnoses being made only 3-10% of the time [14]. However, in patients additionally exhibiting extracardiac abnormalities, diagnostic yield increases considerably (>20%) [14]. Despite the relatively low yield of genetic testing in isolated congenital heart disease, isolated aortic arch anomalies appear to be an exception to this rule, with reports of chromosome 22q11 deletion in up to 24% of subjects [15]. Furthermore, a recent report revealed non-syndromic familial vascular rings associated with duplication in the TBX1 gene [16]. These and other studies will continue to add to the medical literature regarding genetic causes of vascular rings going forward.

Our report represents the first reported coexistence of a double aortic arch and duplication of chromosome 10q11.22q11.23. According to Stankiewicz et al., deletions and reciprocal duplications of 10q11.22q11.23 are most commonly associated with clinical phenotypes of developmental delay and intellectual disability [17]. In this series, there were 24 individuals with microdeletions and 17 individuals with microduplications. This study was limited by the fact that clinical phenotypes were only collected from individuals with microdeletions - as such, the duplication in our patient was judged to be a variant of uncertain significance [17]. While the genetic change observed in our case is perhaps incidental, it is important to document new associations given the expanding field of cardiogenetics.

## Conclusions

Vascular rings represent a relatively rare congenital vascular abnormality, and diagnosis can be delayed due to overlapping clinical symptoms with more common conditions. Seemingly common respiratory and gastrointestinal symptoms should raise concern for a vascular ring if symptoms persist despite treatment. Imaging modalities such as chest radiography and echocardiography are not particularly sensitive, and if a high concern remains after negative or equivocal studies, 3D imaging with cardiac CT or MRI should be obtained. Finally, it is important to be aware of the evolving field of cardiogenetics and indications for testing, as a linked genetic diagnosis can be very helpful for anticipating patient needs over time as well as family counseling.
